# Transactions of the Association of American Physicians

**Published:** 1888-02

**Authors:** 


### Book Reviews.

---

TRANSACTIONS OF THE ASSOCIATION OF AMERICAN PHYSICIANS. Second Session held at Washington, D. C., June 2 and 3, 1887.

The conservative element of this Association is strikingly exemplified in the addition to the seventy-four original members of only seven new ones, two of whom are stars in the profession, while there is but one added to the former list of seven honorary members. As there may be a few more good and true medical men in the sections not yet represented in this select body, it might conduce to the edification of all concerned to fill up the complement allowed in both classes, regular and honorary, during the intervening period, so as to have a better showing from different departments of investigation at the next annual meeting.

In the selection of topics for papers and discussions, the views of this Association are quite as circumscribed as in the choice of members, having a unique rarity in their characteristic manifestations ; and yet there are found among the seventeen contributions three different articles upon that *ignis fatuus* of gaseous enemata in the treatment of phthisis. It is observed that in several of the papers there are terms used which are not ordinarily met with, and which the writer has failed to find in the medical dictionaries at his command, so that it might be well to publish a glossary explaining such terms in the next volume of transactions.

